# Impaired cerebrovascular reactivity after acute traumatic brain injury can be detected by wavelet phase coherence analysis of the intracranial and arterial blood pressure signals

**DOI:** 10.1007/s10877-013-9484-z

**Published:** 2013-06-08

**Authors:** Per Kvandal, Lawrence Sheppard, Svein A. Landsverk, Aneta Stefanovska, Knut A. Kirkeboen

**Affiliations:** 1 Intensive Care Unit, Oslo University Hospital, 0407 Ullevål, Oslo, Norway; 2 Physics Department, Nonlinear Biomedical Physics Group, Lancaster University, Lancaster, UK; 3 Present Address: Division of Ecology and Evolution, Faculty of Natural Sciences, Imperial College London, London, UK; 4 Department of Anesthesiology, Ullevål and Faculty of Medicine, Oslo University Hospital, University of Oslo, Oslo, Norway

**Keywords:** Brain injury, Autoregulation, Cerebrovascular reactivity, Cerebral perfusion pressure, Wavelet transform, Spectral energy, Wavelet phase coherence, Phase shift

## Abstract

The objective of the study was to evaluate the wavelet spectral energy of oscillations in the intracranial pressure (ICP) signal in patients with acute traumatic brain injury (TBI). The wavelet phase coherence and phase shift in the 0.006–2 Hz interval between the ICP and the arterial blood pressure (ABP) signals were also investigated. Patients were separated into normal or impaired cerebrovascular reactivity, based on the pressure reactivity index (PRx). Spectral energy, phase coherence and phase shift in the low frequency and cardiorespiratory intervals were compared for the two groups. Data were prospectively collected and analyzed retrospectively in 22 patients, within the first week after acute TBI. The ICP and ABP signals were continuously recorded for $\cong$40 min and the wavelet transform was used to calculate the spectral energy and phase of the signals. The average ICP wavelet energy spectrum showed distinct peaks around 1.0 (cardiac), 0.25 (respiratory) and 0.03 Hz. Patients with normal cerebrovascular reactivity (negative PRx) had 38.6 % (±SD 16.7 %) of the mean wavelet energy below the lower limit of the respiratory frequency band (0.14 Hz) compared to only 18.1 % (±SD 17.8 %) in patients with altered cerebrovascular reactivity (positive PRx) (difference: *p* = 0.0057). Wavelet phase coherence between the ABP and ICP signals was statistically significant (*p* < 0.05) in the 0.006–2 Hz interval. The phase shift between the ABP and ICP signals was around zero in the 0.14–1.0 Hz interval. Seven patients with PRx between −0.4943 and −0.1653 had a phase shift in the interval 0.07–0.14 Hz, whereas 15 patients with PRx between −0.1019 and 0.3881 had a phase shift in the interval 0.006–0.07 Hz. We conclude that the wavelet transform of the ICP signal shows spectral peaks at the cardiac, respiratory and 0.03 Hz frequencies. Normal cerebrovascular reactivity seems to be manifested as increased spectral energy in the frequency interval <0.14 Hz. A phase shift between the ICP and ABP signals in the interval 0.07–0.14 Hz indicates normal cerebrovascular reactivity, while a phase shift in the interval 0.006–0.07 Hz indicates altered cerebrovascular reactivity.

## Introduction

The cerebral vasculature is continuously adapting to changes in arterial blood pressure (ABP) to maintain a stable cerebral perfusion pressure (CPP). Cerebral perfusion is closely regulated to match the metabolic needs and oxygen demands in the brain tissue. The alterations in cerebral vascular tone change intracerebral blood volume and intracranial pressure (ICP). Perturbations in the ICP signal appear as slow oscillations below the pulsatile components of the heartbeat and respiratory cycle (<0.1 Hz) [1]. In acute traumatic brain injury (TBI) autoregulation of cerebral perfusion is disturbed and care must be taken to maintain CPP at a stable level.

The phase relationship between spontaneous fluctuations in ABP and cerebral blood flow velocity (CBFV) in the median cerebral artery has been studied to evaluate if cerebrovascular reactivity is intact after TBI. A phase shift indicates an active control system [2]. However CBFV cannot be measured continuously to evaluate cerebrovascular reactivity in the acute care of TBI and other non-invasive modalities have been described. The relationship between the averaged means of slow waves of ABP and ICP is termed the pressure reactivity index (PRx). A negative correlation indicates a pressure-active vascular bed with preserved autoregulation, whereas a positive correlation indicates a pressure-passive vascular bed with impaired cerebrovascular reactivity [3]. Moreover, it was shown that PRx reflets changes in cerebral blood flow and cerebral autoregulatory capacity [4]. This suggested a close link between blood flow and ICP in head injured patients.

The mechanism of autoregulation is continuously involved in adjusting ICP, thus resulting in a highly non-linear and non-stationary dynamical process. Consequently, the spectral components of the ICP, with their corresponding amplitudes and phases vary as functions of time. Time–frequency methods have been proposed to study time-variable oscillatory processes. In the present study, the wavelet transform using the Morlet mother wavelet [5, 6], was applied to obtain time-dependent spectral components in the ICP and ABP signals in the 0.006–2 Hz interval.

The relationship between the phases of two oscillatory processes at a specific frequency is defined as the phase coherence. If a characteristic phase difference is maintained between two signals, they have high phase coherence [7]. Recently, a new method that can capture the phase coherence of time-varying processes was proposed [8, 9]. We apply it to obtain phase relationships between the ICP and ABP and investigate the coherence and phase shifts between the two signals at each frequency value in the interval 0.006–2 Hz.

We hypothesized that wavelet transform and wavelet phase coherence analysis of the ICP and ABP signals, providing data of phase shifts throughout the autoregulatory part of the spectrum, can yield a new method to evaluate autoregulation in acute TBI.

Our first aim was to describe the spectral content of the ICP signal in terms of oscillations and wavelet spectral energy. The second aim was to determine the interactions between ICP and ABP in terms of wavelet-based phase coherence and phase shifts in the 0.006–2 Hz interval. The third aim was to compare spectral energy of the ICP signal and phase shifts between the ICP and ABP in the cardiorespiratory and low frequency interval after separating acute traumatic brain injured patients into normal or altered cerebrovascular reactivity based on the PRx index.

## Materials and methods

### Patients

The local ethics committee approved the study. Written informed consent was obtained from the relatives of the patients. Twenty-two patients (14 males, 8 females), mean age 41.6 ± 9.9 years (mean ± SD) with acute TBI admitted directly to the hospital emergency room, were included in the study. Patients with a combination of brain injury and other trauma injuries were also included. The patient characteristics are summarized in Table 1.

**Table 1:** Patient characteristics

GCS mean	5.8 ± 2.4
Polytrauma (n)	7
Isolated head injury (n)	15
Injury mechanism	
Fall (n)	9
Transport accident (n)	13
Neurosurgery	
No surgery (n)	11
Evac. hematoma/contusion	9
Hemicraniectomi	2

*GCS* Glasgow coma scale

### Protocol

Recordings were performed in the intensive care unit (ICU) after initial stabilization with the patients in the supine position and the upper body elevated between 20° and 30° during measurements. The indication for ICP insertion was according to the Brain Trauma Foundation guidelines (Journal of Neurotrauma, Vol 24, Suppl 1). ICP was measured in the right frontal lobe with a strain gauge transducer (Codman, Codman and Shurtleff Inc., Raynham, MA, USA) implanted intraparenchymally. All patients were monitored using standard equipment (Marquette Solar 8000i; GE Healthcare, Bucks, UK), including electrocardiogram, invasive ABP in the radial artery, pulse oximetry and core temperature. The ABP pressure transducer was situated at the level of the earlobe. Measurements were made on day 4.3 ± 3.7 post injury. To assure uninterrupted good signal quality no procedures or movements were allowed during the 37 ± 17 min measurement time. Patients who developed intracranial hypertension were not included. Standard agents of sedation were fentanyl, midazolam and/or propofol. Patients were mechanically ventilated in either a pressure- or volume-controlled mode (Servo^i^, Maquet Critical Care, Solna, Sweden). Physiological variables and patient characteristics are given in Tables 1 and 2.

**Table 2:** Patient characteristics during measurement period

CPP mmHg	67.9 ± 7.6
ICP mmHg	12.3 ± 4
MAP mmHg	80.3 ± 8.5

Core temperature (°C)	36.4 ± 0.58

Arterial CO_2_ (kPa)	Patients
4–4.4	4
4.5–4.9	9
5.0–5.4	6
5.5–5.9	2
Unknown	1
Norepinephrine	19
No vasopressor	3
Ventilator mode	
Controlled	20
Support	2

*CPP* cerebral perfusion pressure, *MAP* mean arterial blood pressure, *ICP* intracranial pressure, °C degree Celsius, *kPa* kiloPascal

### Data acquisitions and analyses

Data were transferred from the analog output of the Marquette monitor to an analogue-to-digital converter (NIDAQ, National Instruments, Austin, TX, USA) and then to a laptop using data acquisition software (VI logger, National Instruments). The sampling frequency was 100 Hz.

### Wavelet transform and wavelet-based phase coherence analysis

**Fig. 1:**
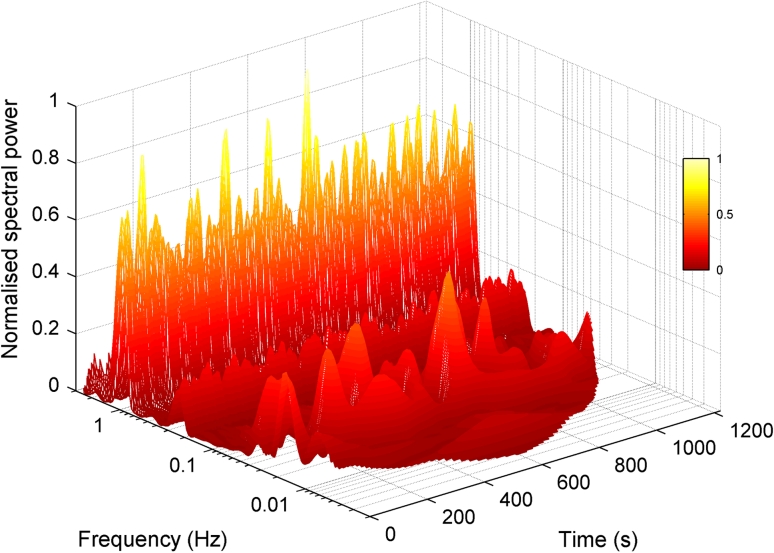
Wavelet transform magnitude showing the frequency content of the ICP signal in the interval 0.006–2 Hz for a subject with normal autoregulatory activity

After resampling to 10 Hz using a moving average, the Morlet wavelet transform [5] was applied to both the ABP and ICP signals, as described by Stefanovska et al. [6]. The wavelet has a centre frequency equal to one over its scale, and a scale increment of 5 % resulting in logarithmic frequency resolution. Both the frequency resolution and the time resolution (equivalent to the size of the wavelet in the time domain) are thus scaled appropriately to each frequency. The wavelet transform of a signal has an amplitude and phase for each frequency component. A wavelet energy spectrum can be calculated from the squared modulus of the wavelet amplitudes, and time-averaged to produce an energy or power (energy per second of signal) spectrum. Since the units of the ICP signal are mmHg the units of signal power are mmHg^2^/s. An example of the wavelet energy spectrum of an individual patient is given in Fig. 1.

Corresponding frequency components from the two signals can be compared by checking for significant phase coherence between the phase time series, as described by Bandrivskyy et al. [8] and Sheppard et al. [9]. If there is a fixed phase relationship between the oscillations at a given frequency in the two signals, the wavelet phase coherence can be as high as 1. A low value of wavelet phase coherence indicates that no particular phase relationship exists, and there is no relationship between the oscillations in the two signals at this frequency. In order to test the wavelet phase coherence values obtained, we used the method of surrogates, as described by Schreiber and Schmitz [10]. By generating amplitude-adjusted Fourier transform surrogates of each signal, and calculating a phase coherence value for each surrogate pairing, we can generate a test distribution of wavelet phase coherence values between signals in which any relationship between ABP and ICP signals has been destroyed by the mathematical operation of phase shuffling [11]. If the actual value of wavelet phase coherence is higher than the 95th ‰ of phase coherence values obtained for this artificial unrelated surrogate distribution, the phase coherence value is high enough to indicate a significant relationship between the signals at this frequency.

If the phase coherence value is significant, then a particular phase difference is being maintained between the ABP and ICP signals at this frequency. We can plot the significant phase difference values as a function of frequency, to see how the phase relationship between oscillations in ABP and oscillations in ICP depends on the frequency of those oscillations.

We introduce the term phase agreement to indicate the similarity between phase difference values determined for different subjects. The phase difference for each subject can be represented as a unit phasor, and the phasors combined in the complex plane. If the magnitude of the mean phasor is large the agreement about the mean phase is good; if it is small then the subjects do not agree on any particular phase difference at that frequency.

### Cut-off frequency of the phase shift

Having observed an obvious phase shift at high frequencies in seven subjects, and at lower frequencies in others, we introduce the following method to determine the frequency at which the phase relationship between the wavelet components of the ICP and ABP transforms changes. After finding the phase difference between ABP and ICP at each frequency, we choose a shift frequency *f*. Phase values corresponding to frequencies below *f* are rotated back by 180°, and those above are not. Then, the average cosine of all these phase values is obtained. Next, the maximum of this quantity as it varies with *f* is found: at the maximum, phase values above *f* are near zero and those below *f* are near antiphase. Thus, *f* is the cut-off frequency. In one case with high PRx value no cut-off was found and *f* was set to the lowest frequency in the transform.

### PRx index

The PRx was introduced by Czosnyka et al. [3] and is essentially a cross correlation measure between the variation in ABP and ICP, considered inside a particular frequency interval. Frequencies above 0.2 Hz and below 0.005 Hz are removed by use of a moving average and windowing (of 5 and 200 s respectively), and the cross correlation is computed. Where the value obtained is negative, this is deemed to indicate that the reactivity of the vascular bed is preserved [1, 13]. We used as many non-overlapping 200 s windows as would fit within each recording to estimate the mean PRx and standard error of mean for each subject.

### Statistical analysis

SPSS software was used to perform the descriptive statistics. Programs written in MatLab (MatWorks) were used to perform the mathematical operations of the wavelet transform, wavelet-based phase coherence and the generation of the surrogate distribution used to test for significant coherence. Where statistics were compared between groups of subjects, the unpaired nonparametric Kolmogorov–Smirnov test was applied to test for significant differences between the groups. Data are given as mean ± SD. A *p* value of <0.05 was considered statistically significant.

## Results

### Spectral content of the ICP signal; frequency peaks, energy of the wavelet spectrum and relation to the pressure reactivity index (PRx)

**Fig. 2:**
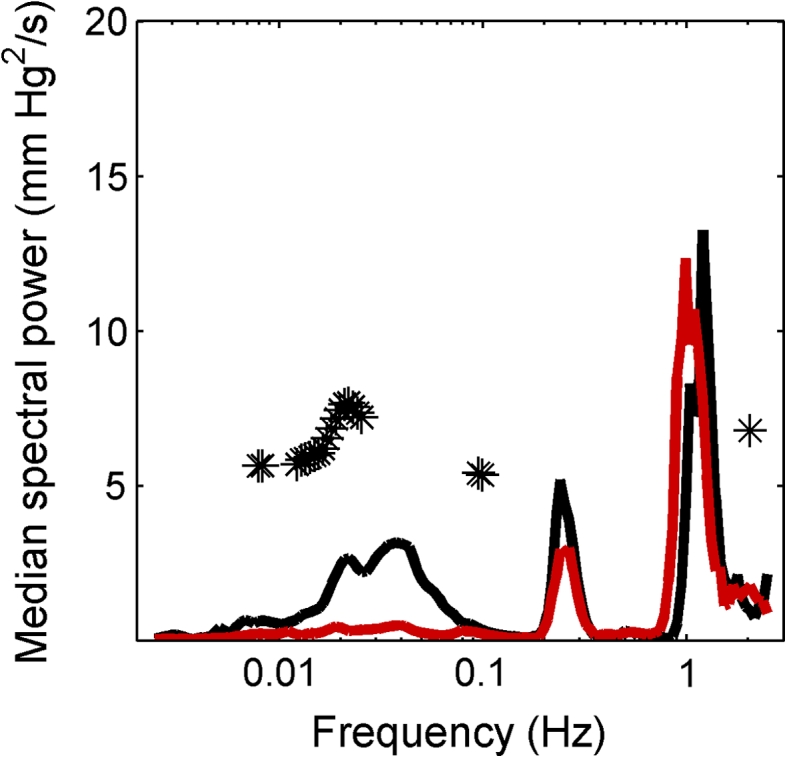
The median wavelet spectra of the 22 patients after separation into a group with normal (*black*) and altered (*red*), autoregulatory activity. The separation was made according to the pressure reactivity (PRx) measure. Significant difference between groups is indicated with *asterisk*

Average ICP wavelet energy spectrum for all patients showed three distinct peaks (Fig. 2). In the interval from 0.14 to 2.0 Hz a cardiac peak at around 1.0 Hz and a respiratory peak at 0.25 Hz were found. The interval 0.06–0.14 Hz had a frequency peak at around 0.03 Hz.

The PRx mean values and their standard errors are summarized in Table 3. Nine patients had a negative PRx, with a mean of −0.2827 (±SD 0.1489). Thirteen patients had a positive PRx, with a mean of 0.2020 (±SD 0.1389). The mean wavelet energy normalized by signal length of the whole spectrum from 0.006 to 2 Hz was 0.052 (±SD 0.074) V^2^/s. The proportion of the wavelet energy below 0.14 Hz was 26.49 (±SD 19.85 %) for all patients. Patients with a negative PRx had significantly higher mean wavelet energy <0.14 Hz compared to patients with a positive PRx [38.6 % (±SD 16.7 %) vs. 18.1 % (±SD 17.8 %) *p* = 0.0057, Fig. 2].

**Table 3:** The PRx mean value and standard error for each patient

Mean PRx	SE
−0.373	0.073
0.053	0.090
0.087	0.083
0.345	0.050
−0.165	0.104
−0.494	0.078
0.377	0.042
−0.437	0.102
−0.258	0.074
−0.102	0.094
0.073	0.064
−0.358	0.051
0.379	0.044
−0.293	0.037
0.261	0.048
0.064	0.055
0.193	0.034
0.030	0.084
0.258	0.064
0.118	0.045
−0.064	0.048
0.388	0.057

### Frequency dependence of wavelet phase coherence and phase shifts between the ABP and ICP signals

**Fig. 3:**
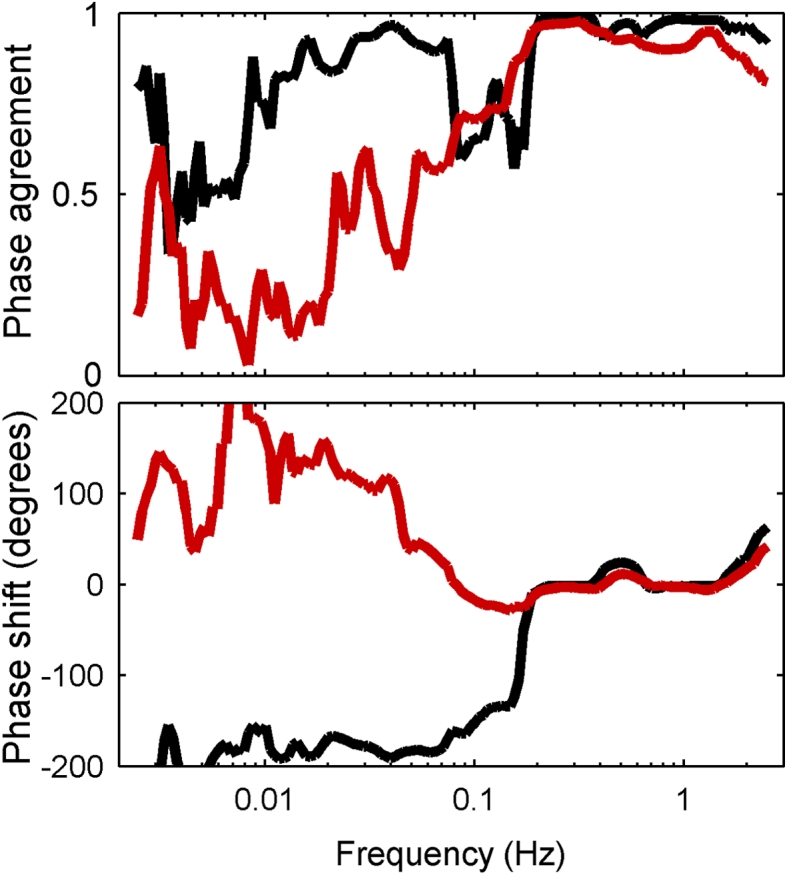
Phase agreement (*upper panel*) and average phase shift (*lower panel*) for the whole group of subjects divided into two groups, depending on whether the phase shift between ABP and ICP is completed (to −180°) in the 0.07 frequency interval (*black*) or at lower frequencies (*red*). Patients whose phase shift occurs within the frequency range 0.07–0.14 Hz (*black*) have preserved autoregulation according to their pressure reactivity index (PRx). For the other group (*red*), in which the phase shift developed over a wider frequency range and in the opposite direction (towards +180°), the PRx values indicated lost autoregulation. Note that a phase of −180° is the same as a phase of +180°. Phase values are presented so as to minimize discontinuities between neighbouring values

**Fig. 4:**
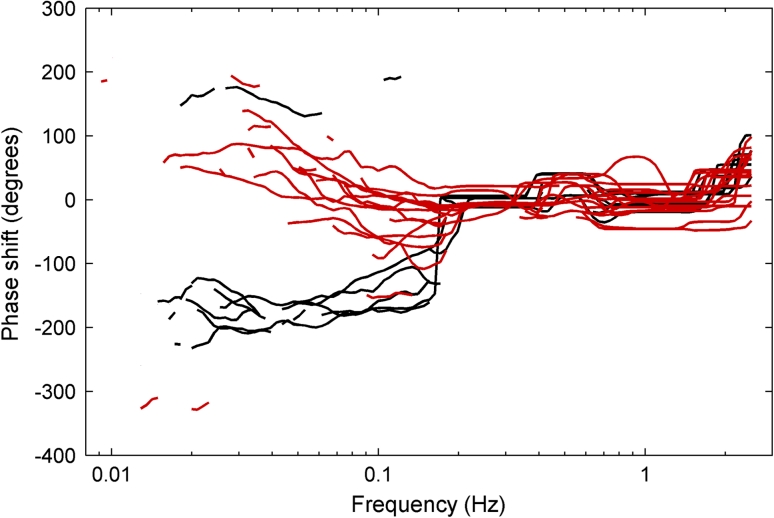
The phase difference between arterial blood pressure (ABP) and intracranial pressure (ICP) for the 22 patients plotted where the wavelet phase coherence between the ABP and ICP signals is found to be significant for the individual patient. The patients marked *black* show a clear phase shift and go into an antiphase relationship between 0.07 and 0.2 Hz. The patients marked *red* show phase change that develops over a wider frequency range and extents to 0.006 Hz

The wavelet-based phase coherence between the ABP and ICP signals was around 1 in the cardiorespiratory interval (0.14–2.0 Hz). In the 0.006–0.14 Hz interval coherence showed more variability, but remained statistically significant (*p* = 0.05) compared to surrogate data for all patients (Fig. 3, upper panel). The phase shift between the ABP and ICP signals was around zero in the 0.14–2.0 Hz interval. In the 0.006–0.14 Hz interval patients with positive PRx had a phase shift of approximately π radians (180°) (Fig. 3, lower panel). Patients could be divided in two groups according to the frequency at which the phase shift became apparent. Seven patients had an obvious phase shift in the 0.07–0.14 Hz interval. These patients had PRx from −0.4943 to −0.1653. Fifteen patients had a phase shift in the 0.006–0.07 Hz interval. These patients had PRx from −0.1019 to 0.3881. In Fig. 4 the phase shifts for the individual patients are plotted.

**Fig. 5:**
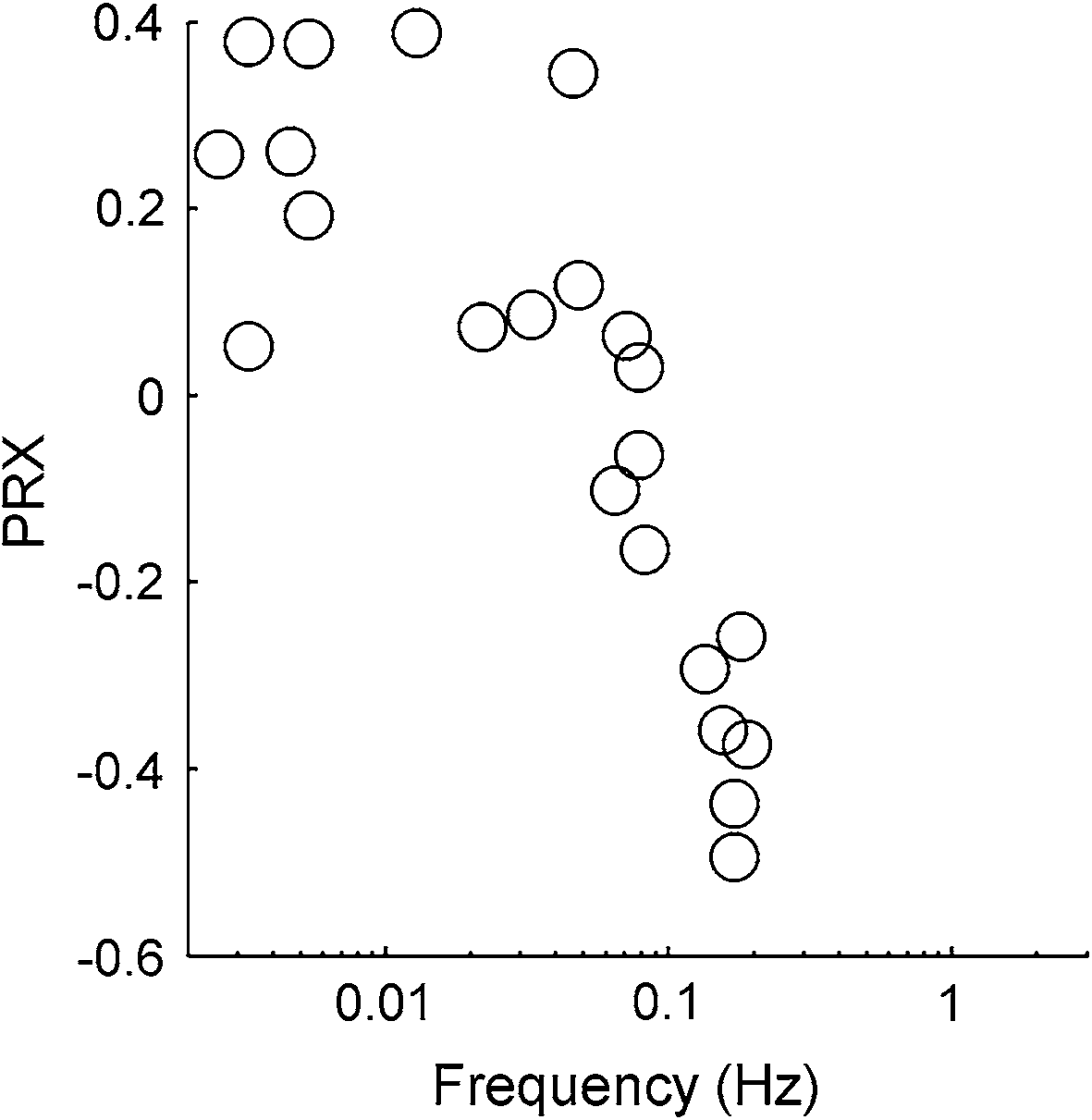
The relationship between the cut-off frequency *f* at which wavelet components go into antiphase and the PRx index for each individual subject. The relationship is strongly significant with a correlation coefficient of *r* = −0.91 (*p* = 3 × 10^−9^ obtained by *t* test)

We determined a cut-off frequency for every subject as described in the methods. The relationship between the cut-off frequency and the PRx index for each individual subject is presented in Fig. 5. It indicates a strongly significant (Pearson’s correlation coefficient *r* = –0.91 with *p* = 3 × 10^−9^ obtained by Student’s *t* test) relationship between PRx and the cut-off frequency *f* at which wavelet components go into antiphase.

## Discussion

In this study wavelet transforms of the ABP and ICP signals were performed and the wavelet-based phase coherence was calculated in patients with acute TBI. Spectral peaks corresponding to cardiac, respiratory and a lower frequency (around 0.03 Hz) were found in the ICP signal. Cerebrovascular reactivity was manifested as increased spectral energy in the frequency interval <0.14 Hz. Preserved cerebrovascular reactivity was indicated by a phase shift between the signals in the 0.07–0.14 Hz interval. A different pattern was obtained in patients with impaired cerebrovascular reactivity, where a phase shift was found in the frequency interval <0.07 Hz.

**Fig. 6:**
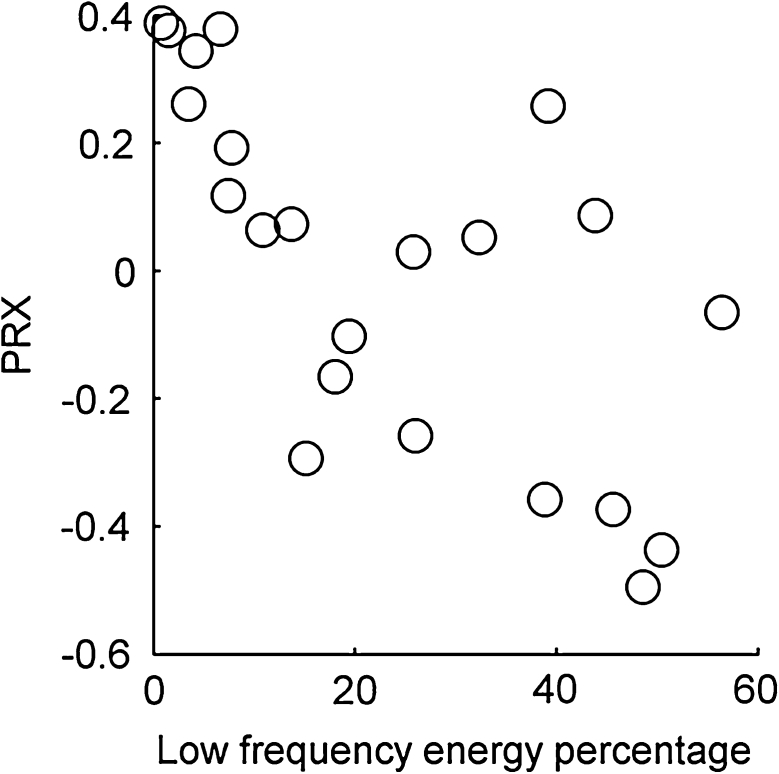
A *scatter plot* showing the significant correlation of *r* = −0.68 (*p* = 5 × 10^−4^ obtained by *t* test) between the proportion of signal energy in the low frequency (<0.14 Hz) regime, and the PRx

High spectral energy in antiphase with ABP fluctuations requires active cerebrovascular reactivity. This activity in the negative PRx group produced higher energies than mechanical transmission of ABP fluctuations in the positive PRx group. Severe impairment and very high PRx would probably result in high energy too, but in phase with ABP fluctuations. The scatter plot in Fig. 6 illustrates the significant correlation between the proportion of signal energy in the low frequency (<0.14 Hz) regime, and the PRx.

### Methodological considerations

Previous studies have found oscillatory components in the 0.008–0.05 and 0.05–0.15 Hz frequency interval [12]. The latter are called M-waves and occur independently of respiration in many different organs, including brain perfusion signals. Oscillations in the 0.008–0.05 Hz interval found in pathological states of brain perfusion are termed B-waves.

The spectral content of the B-wave interval can be determined either in the frequency or in the time–frequency domain. The wavelet transform is a time–frequency method that facilitates the analysis of biological signals containing low frequencies that are non-stationary. This is because it provides continuous rather than discrete representation of the frequency content. Secondly, it provides logarithmic frequency resolution, which is particularly important at low frequencies in systems that encompass a wide range of frequencies. In a study of oscillations in skin perfusion measured with laser Doppler flowmetry (LDF), the frequency content as evaluated by use of the wavelet and Fourier transforms was compared in the interval 0.005–2 Hz [14]. The low frequency content appeared as distinct peaks only when the wavelet transform was applied.

Fluctuations in pCO_2_ and the level of sedation amount to confounding variables when measuring cerebrovascular reactivity. They affect cerebrovascular diameter and reduce sympathetic drive to the vasculature. In addition, CPP must be kept constant and within the range where perfusion of the injured brain is autoregulated. In order to minimize the effect of extraneous factors the levels of sedation, pCO_2_ and CPP were kept stable (Table 2) during the measurements.

In the present study the PRx index was used to separate patients with normal and impaired cerebrovascular pressure reactivity. A negative correlation indicates a pressure active vascular bed with preserved cerebrovascular reactivity, whereas a positive correlation indicates a pressure passive vascular bed with impaired reactivity. In the study by Czosnyka et al. [3] outcome was related to the average PRx for the whole period of intensive care monitoring. Patients with a PRx <–0.2 experienced a favourable outcome while the outcome was unfavourable in 82 % of patients with a value >0.2. In the group with a PRx between –0.2 and 0.2 the outcome of favourable versus unfavourable was 55 versus 45 %. The index represents a graded loss of cerebrovascular reactivity—not just present or absent [15]. Because of the relatively small sample size and lack of outcome data in the present study we grouped the patients depending on whether the regression line was positive or negative. The relatively short data sampling time of around 37 min and the grouping of patients into normal or impaired cerebrovascular reactivity based on this short time period appeared to be a weakness of the study compared to the long data series conventionally recorded in ICUs. However, the data used here were of higher quality than usual in that they were almost free of transients and artefacts. All patients were in the supine position, with no other activity going on than just the measurements; e.g. no change in ventilation, level of sedation, infusion of vasoactive drugs or blood samples drawn from the arterial line. Provided that a period of at least 20-min of such data are available, the analysis is statistically valid down to the lowest oscillation frequency of interest (0.01 Hz).

We aimed that the group of patients to be included should be clinically uniform, viz. with acute TBI defined by a score of 8 or below on the Glasgow coma scale (GCS) and in need of relatively standardized treatment at the ICU. Patients who develop intracranial hypertension are more deeply sedated, are treated with hypertonic fluids to reduce brain edema, need more vasopressors to keep a stable CPP, and are sometimes transiently hyperventilated. Patients with elevated ICP are more likely to have impaired cerebrovascular reactivity and a positive PRx. By excluding this latter group, the range of PRx values is probably narrowed and the power of the statistical analysis reduced. In future studies a comparison between patients with acute TBI with and without high ICP would be of great interest. Additionally, important information could be generated by comparing cerebrovascular reactivity during episodes of high ICP versus normal ICP in the same patients.

### Energy of oscillations in the ICP signal

The analysis of slow waves with logarithmic resolution to evaluate vascular activity has not, as far as we are aware, previously been applied in studies of cerebral autoregulation. Wavelet transform enabled identification of low frequency component in the ICP signal peaking at around 0.03 Hz. The oscillatory activity in this frequency interval is generated by vasomotion, the rhythmic changes in vascular diameter during normal states of perfusion. These changes are mediated by both, the rhythmic activity of smooth muscle cells and by neurogenic regulation. We hypothesize that a reduced level of autoregulatory vasomotion caused by severe acute TBI can explain the lower median spectral energy in the group of patients with altered cerebrovascular reactivity.

The characteristic changes in spectral energy observed in our study can be associated with changes in magnitude of slow waves reported previously. Balestreri et al. [13] observed that in the B-wave frequency range the ICP magnitude of slow waves was decreased in severely brain injured patients who died compared to those who survived. However, the study did not provide an explanatory model for this observation.

It has also been shown that a change in slow waves of the ICP signal in TBI indicates an autoregulatory response to changes in cerebral perfusion [16]. B-waves have also been shown to occur simultaneously with vascular oscillations in an experimental study with incremental ICP increases [17].

A loss of neural vascular control might possibly be able to explain the difference between patients with normal or altered cerebrovascular reactivity. Lang et al. [18] compared normal and comatose subjects of various etiologies. They measured bilateral flow in the cerebral medial artery concomitantly with blood perfusion in the fingertips, evaluated by LDF, and ABP. Comatose patients had profound reductions in the amplitude of B-waves in peripheral blood perfusion and in ABP, compared to normal subjects, suggesting a severe impairment of the sympathetic pathway. In a separate study of skin blood perfusion recorded by LDF the wavelet transform was used to show that oscillations in the B-wave frequency range (0.03 Hz) are dependent on sympathetic nerve activity [19].

### Wavelet-based phase coherence and phase shift

We found high coherence and no phase shift between the ICP and ABP signals in the 0.14–1.0 Hz frequency interval implying direct hydrostatic transmission to the ICP of the pressure waves caused by heartbeat and respiration. However, we also found significant phase coherence between the MAP and ICP signals in the M-wave interval. Whereas autoregulation reduces the coherence between blood flow and ABP (rendering perfusion independent of changes in systemic blood pressure), we hypothesized that autoregulation would actually increase phase coherence between the ICP and ABP signals, manifested as a strong negative correlation in the PRx measure.

From the phase shifts plot we found that the patients could be divided in two groups according to where in the M-waves interval the phase shift became apparent. Patients with greatest cerebrovascular reactivity (measured by PRx) showed a phase shift at higher frequencies, whereas patients with altered cerebrovascular reactivity showed a phase shift in the B-waves interval. Thus, it seems that autoregulatory activity is a higher frequency process (faster adaptation) in patients with greatest autoregulatory activity overall. In a study that measured the correlation between CBFV and ABP in normal subjects and in patients with TBI, the correlation between these variables was non-significant in normal subjects and significant in 21 patients with impaired cerebral vasomotor reactivity [20]. This is consistent with our wavelet data where ICP and ABP are in antiphase in patients with a negative PRx; ICP changes reflect changes in cerebrovascular volume rather than flow.

In a study by Latka et al. [21] patients with initial GCS 3–8 caused by TBI or vascular pathology were included. Complex wavelet transforms between the ABP and ICP signals were analyzed to study the phase difference between the two time series in the B-waves interval. The case mix in the study and the small number of patients in each group indicates that the outcome data should be interpreted carefully in the clinical setting of TBI. The time averaged synchronization index was proposed as a quantitative measure of the stability of the phase difference between the ABP and ICP fluctuations. A low value of synchronization reflected a normal reactive vascular bed while a high value indicated pathological entrainment between the ABP and ICP fluctuations. In the present study the discriminating feature was whether the coherence occurred in phase or in antiphase. The two studies are mutually complementary.

The phase shift angles between CBFV in the cerebral median artery and ABP were evaluated in subjects after subarachnoidal hemorrhage during slow oscillations under the 6 ventilations/min protocol [22]. A phase difference between slow waves of CBFV and ABP of >30° indicated preserved autoregulation. The phase shift method was shown to be an effective tool to assess autoregulation. However, the slow breathing protocol cannot be performed continuously. Evaluating the phase shift continuously without the need for perturbation has obvious advantages.

The wavelet-based phase coherence analysis indicates that intrinsic oscillations in ICP at low frequencies are interfering with oscillations in ABP in patients with less severe TBI. This interference could be resulting from autoregulatory processes, which counteract the variation in ABP. The characteristics of this interference may be additionally evaluated by analysing the phase shift between ABP and ICP signals and the frequency at which it occurs.

## Conclusion

The wavelet transform of the ICP signal shows spectral peaks at the cardiac, respiratory and the 0.03 Hz frequencies. Normal cerebrovascular reactivity seems to be associated with an increased spectral energy in the frequency interval <0.2 Hz, in antiphase with the oscillations of blood pressure. The results suggest that the M-wave frequency interval (0.06–0.15 Hz) may be used to discriminate between patients with normal or altered cerebrovascular reactivity: a phase shift between the ICP and ABP signals in the interval 0.07–0.14 Hz indicates normal cerebrovascular reactivity, while a phase change that extends over the interval 0.006–0.07 Hz indicates altered cerebrovascular reactivity. The spectral energy and phase shift, as obtained by wavelet transform and wavelet-based phase coherence analysis, might at a later stage be developed into clinical variables for surveillance of autoregulation in patients with TBI.

## Appendix

We applied the continous Morlet wavelet transform operation using the simplified Morlet wavelet described in [23], using a mother wavelet with centre freguency *f*_0_ set equal to 1, rescaled in 5 % increments from 0.0025 to 2.5 Hz.

The wavelet transform operation produces a matrix of complex numbers representing the amplitudes and phases of oscillations with different frequencies at different times. The spectral energy of these oscillations is obtained by squaring (normalized by scale) the amplitudes, producing a scalar quantity which can be summed over all frequencies and time-averaged. In the case of an ICP signal in mmHg for example, this gives a signal power in mmHg^2^/s.

The phase of each wavelet component can be compared with the phase of the corresponding component from the transform of another signal, such as the ABP signal. The phase difference between the components at a given frequency is *θ*_*t*_ and varies as a function of time *t* (between 1 and *T*). The mean phasor *P* is given by

$$ P = \frac{1}{T}\sum\limits_{t = 1}^{T} {e^{{i\theta_{t} }} } $$

The phase coherence is equal to the amplitude of *P*. If it is high the subject preserves a particular typical phase difference between the wavelet transforms at this frequency.

The typical phase difference $\varphi$ is the phase of *P*. Subject *n* (of *N* subjects) has a typical phase difference $\varphi_{n}$. Now we find the average *M* over all subjects,

$$ M = \frac{1}{N}\sum\limits_{n = 1}^{N} {e^{{i\varphi_{n} }} } $$

We define the phase agreement to be the amplitude of *M*. If it is high the subjects agree about the typical phase difference between their ICP and ABP wavelet transforms at this frequency. The phase shift on which they agree is given by the phase of *M.*

The use of a distribution of AAFT surrogate values to determine the statistical significance of the phase coherence between ICP and ABP at every frequency of the wavelet transform is described in [9].

Having determined the statistical significance of the phase coherence, the phase difference between the signals can be plotted.

Having determined the phase difference associated with each subject at a given frequency, the phase agreement of a group of subjects, and the phase difference upon which they agree, can be plotted.
